# The gut microbiota: A new perspective for tertiary prevention of hepatobiliary and gallbladder diseases

**DOI:** 10.3389/fnut.2023.1089909

**Published:** 2023-02-06

**Authors:** Xiaoyu Huang, Yi Yang, Xueli Li, Xiaoya Zhu, Dan Lin, Yueran Ma, Min Zhou, Xiangyi Cui, Bingyu Zhang, Dongmei Dang, Yuhong Lü, Changwu Yue

**Affiliations:** ^1^Yan’an Key Laboratory of Microbial Drug Innovation and Transformation, School of Basic Medicine, Yan’an University, Yan’an, Shaanxi, China; ^2^Shaanxi Key Laboratory of Chemical Reaction Engineering, College of Chemistry and Chemical Engineering, Yan’an University, Yan’an, Shaanxi, China

**Keywords:** gut microbiota, hepatobiliary, gallbladder principal diseases, gut–liver–biliary axis, biotherapy

## Abstract

The gut microbiota is a complex ecosystem that has coevolved with the human body for hundreds of millions of years. In the past 30 years, with the progress of gene sequencing and omics technology, the research related to gut microbiota has developed rapidly especially in the field of digestive system diseases and systemic metabolic diseases. Mechanical, biological, immune, and other factors make the intestinal flora form a close bidirectional connection with the liver and gallbladder, which can be called the “gut–liver–biliary axis.” Liver and gallbladder, as internal organs of the peritoneum, suffer from insidious onset, which are not easy to detect. The diagnosis is often made through laboratory chemical tests and imaging methods, and intervention measures are usually taken only when organic lesions have occurred. At this time, some people may have entered the irreversible stage of disease development. We reviewed the literature describing the role of intestinal flora in the pathogenesis and biotherapy of hepatobiliary diseases in the past 3–5 years, including the dynamic changes of intestinal flora at different stages of the disease, as well as the signaling pathways involved in intestinal flora and its metabolites, etc. After summarizing the above contents, we hope to highlight the potential of intestinal flora as a new clinical target for early prevention, early diagnosis, timely treatment and prognosis of hepatobiliary diseases.

**GRAPHICAL ABSTRACT:**
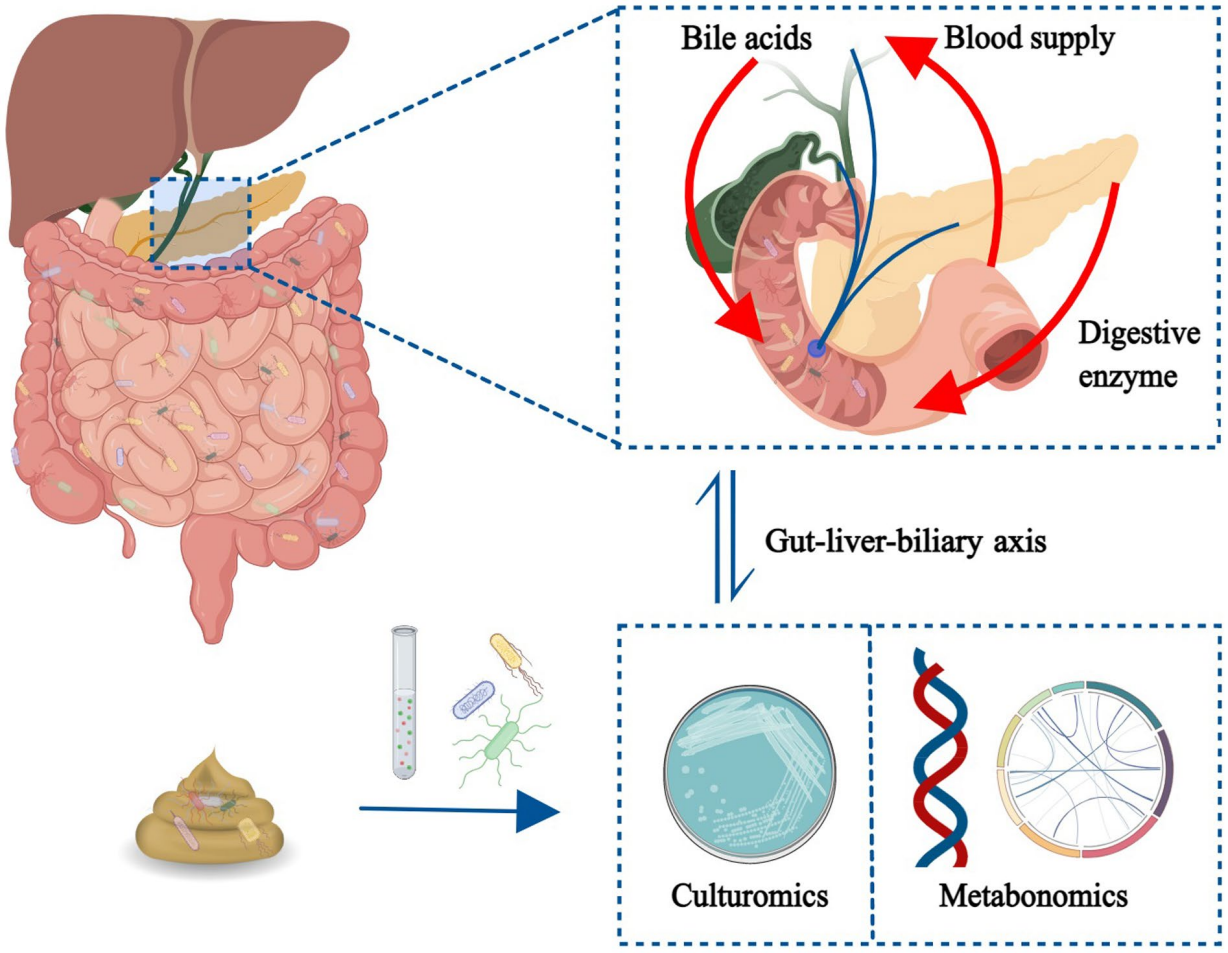

## 1. Introduction

The gut microbiota is a huge microbial community living in the host’s gut and includes a large number of bacteria and a small number of archaea and fungi. The ratio of bacteria to human cells (including red blood cells) in healthy adults has been updated to nearly 1:1 in 2016 (1). The human body and trillions of microorganisms, a dynamic equilibrium system of mutual influence and coevolution, coexist. The gut microbiota has the highest density and has attracted attention because of remarkable clinical significance (2). The Human Microbiome Project, The Human intestinal metagenome Project, and other intestinal microbiota research projects have been carried out. Since the migration of humans to the birthplace of Africa, gut bacteria have evolved, showing racial specificity especially in bacteria that rely on human intestinal conditions (3). The human gut microbiota begins to be acquired from the rupture of the amniotic sac during fetal period. Different delivery and feeding methods affect the colonization of the microbiota, and the diversity and richness of the microbiota form and stabilize in early childhood (4). In addition, gender, age, dietary structure, lifestyle, and other factors affect the structure of gut microbiota. Compared with that of young people, the gut microbiota of the elderly even shows higher diversity and health degree, which is speculated to be a good foundation laid by the previous generation in early life and continues to old age (5). The human gut microbiota can be divided into three intestinal types, i.e., *Bacteroides* (enterotype 1), *Prevotella* (enterotype 2), and *Ruminococcus* (enterotype 3), in accordance with the dominant genus (6). The intestinal type can maintain its stability for a certain period and is difficult to transform. However, long-term environmental influences and dietary changes may result in intestinal-type transformation (7).

At present, the research methods of gut microbiota primarily include multi-omics data integration analysis (based on meta-omics, metagenomics, macrotranscriptomics, proteomics, and metabolomics) and culturomics (based on high-throughput bacterial isolation and culture technology) (8). In recent years, the development of sequencing technology has made substantial progress in omics research. Culturomics, with its advantages of low detection threshold and research depth to the strain level, has isolated and cultured a variety of human gut microbiota for the first time and has been flourishing (9). The two research methods have their own advantages and disadvantages in many aspects, and complementary development can promote the research of human gut microbiota. At the same time, current sampling methods should be improved, or new methods should be designed to improve sample accuracy and bacterial culture technique need to be optimized (10, 11).

## 2. Gut microbiota and hepatobiliary principal diseases

### 2.1. Viral hepatitis

Viral hepatitis is a kind of infectious disease caused by a variety of hepatitis viruses. The internationally recognized liver hepatitis viruses are HAV, HBV, HCV, HDV, and HEV. The hepatitis B caused by HBV has extremely high incidence worldwide, easily progresses into chronic hepatitis, and further deteriorates to liver cirrhosis and liver cancer (12–14). HBV infection manifested as transient infection in BALB/c mice and chronic infection in C57BL/6 mice. A research teams used antibiotics to remove the intestinal flora of BALB/c mice and transplanted fecal microbiota from C57BL/6 mice into BALB/c mice, and found that BALB/c mice also showed immune tolerance to HBV and chronic infection after transplantation. The outcome of HBV infection is highly related to the structure of intestinal flora (15). A clinical study found that patients with HBV-induced chronic liver disease have intestinal dysbacteriosis. The main manifestations are increased potential pathogenic bacteria, such as *Klebsiella*, *Escherichia coli*, *Proteus*, and *Enterobacter*, and decreased beneficial bacteria, such as *Clostridium*, *Ruminococcus*, and *Bifidobacterium* (16). However, another study showed that *Lactobacillus*, which are considered probiotics, are positively associated with the development of chronic hepatitis B, suggesting their contribution to the development of the disease (17). An experiment showed that intestinal flora failure can cause damage to intestinal barrier function and induce the transfer of living commensal bacteria from the gut to the liver (18). Translocated bacteria and their components, such as lipopolysaccharide, can activate hepatic inflammatory cells, promote the secretion of inflammatory factors, inhibit immune response, and prolong the infection time of HBV. At the same time, bile acids (BAs), which are important components with bactericidal and regulatory effects in the intestine, are dysregulated in the middle and late stages of chronic hepatitis B. The main manifestations are increased levels of serum total BAs and primary BAs and significantly decreased levels of fecal and secondary BAs. Intestinal microbiota analysis showed that changes are related to decreased abundance of bacteria involved in BA metabolism (19). The intestinal microbiota dysregulation affects the metabolism of BAs in the intestine, thus blocking the BA circulation pathway and further increasing burden on the liver. For hepatitis A, C, and E, clinical studies showed different degrees of intestinal microbiota disorders in infected patients (20–22). In the early stage, patients with chronic hepatitis C showed abnormal bile acid metabolism. It was found that the transcription level of CYP8B1, a key enzyme in the process of cholic acid biosynthesis, was reduced, and deoxycholic acid in feces was reduced, which was speculated to be mediated by intestinal flora imbalance (23). Reduced diversity, Lactobacillus acidophilus and lactic acid levels have been found in patients (23). Most of the studies on intestinal microbiota related to liver disease progression used 16SrRNA sequencing technology. Although the survival status of bacteria is unknown, 16SrRNA sequencing technology promoted the horizontal study of intestinal microbiota efficiently and accurately. However, longitudinal studies on the causal and progressive relationship between intestinal microbiota and viral hepatitis, such as specific metabolic pathways, remain limited. In the future, more macrotranscriptomics, proteomics, metabolomics, and culturomics methods should be added for further research to provide a solid theoretical basis for new treatment methods, such as fecal bacteria transplantation, and guidance for determining the appropriate donor.

### 2.2. Non-alcoholic fatty liver disease

Nonalcoholic fatty liver disease (NAFLD) is a cumulative metabolically damaging liver disease characterized by the excess of liver free fatty acids and abnormal increase in fat synthesis. In 2021, the Latin American Association for the Study of the Liver issued a statement renaming this disease metabolically associated fatty liver disease (MAFLD) (24). In the same year, the American Association for the Study of Liver Diseases objected to the renaming because of terminology ambiguity and lack of rigor (25). According to statistics, NAFLD has become the most prevalent chronic liver disease in China and a serious threat to human health (26). Without intervention, NAFLD will gradually develop into non-alcoholic fatty liver disease (NAFL), non-alcoholic hepatitis (NASH), NAFLD-cirrhosis, and liver cancer. Some studies showed that the dysregulation and decreased diversity of gut microbiota in patients with NAFLD are related to the progression of NAFLD and BMI (27). The risk is high in patients who are not obese. A study used mouse models to demonstrate the ability of pathogenic *Klebsiella* to induce NAFLD directly, which is associated with endogenous alcohol overproduction (28). The dysregulation of gut microbiota and intestinal barrier damage accelerate the translocation of bacteria and their derivatives, promote the activation and proliferation of liver B cells, and aggravate the inflammatory response of NASH (28). Fortunately, a randomized trial in patients with NAFLD showed that taking probiotics and prebiotics for a year significantly alters gut microbiota diversity and increases the number of probiotics, such as *Bifidobacterium* (29). Indole-3-propionic acid significantly reduces the abundance of *Bacteroides*, *Streptococcus,* and other pathogenic bacteria in the intestine of patients with NAFLD and promotes the repair of intestinal mucosa and barrier (30). Astragalus polysaccharide and chlorogenic acid have been shown to have anti-NAFLD effects in regulating gut microbiota homeostasis (31, 32). The supplementation of ursodeoxycholic acid can increase the relative abundance of *Firmicutes*, reduce the relative abundance of Bacteroides, and partially restore the intestinal microbiota imbalance in all stages of NAFLD (33). The GLP-1 analog Liraglutide and FGF19 analog Aldafermin have ameliorated intestinal microbiota dysregulation in NAFL and NASH, respectively (34, 35). Combined with plant lactobacillus, the dama polysaccharide, which improves insulin resistance, can also significantly alleviate fat degeneration and improve the NAFLD progress (36). Physical exercise has also been shown to improve gut microbiota dysregulation and restore impaired BA metabolism in patients with NAFLD (37). The current research direction is to use probiotics and their metabolites, plant extracts, BA metabolites, gut hormone analogs, behavioral intervention, and other methods alone or in combination to improve the dysregulation of gut microbiota and then delay and control the progression of NAFLD and NASH.

### 2.3. Alcoholic fatty liver disease

Alcoholic fatty liver disease (AFLD) is the steatosis of the liver caused by long-term excessive alcohol consumption, which can develop into alcoholic hepatitis, liver fibrosis, cirrhosis, and even liver cancer. The intestinal microbiota analysis of AFLD mice showed that the abundance values of *Enterococcus, Streptococcus,* and *Enterobacterium* increase especially *Enterococcus*. At the same time, the serum level of LPS increases (38). An experiment showed that with the progress of AFLD, intestinal microbiota imbalance gradually increases, and species richness decreases (39). In mice with alcohol-induced alcoholic hepatitis, early changes in gut microbiota are found to decrease the abundance of *Akkermansia* (40). In alcoholic cirrhosis period, the number of *Streptococcus* and *Enterobacteria* is significantly increased, whereas the number of *Ruminococcus* is significantly decreased. Moreover, the abundance of *Streptococcus* has been shown to be positively correlated with the severity of hepatocyte injury (39). In addition, an experiment showed that the intestinal flora related to BA metabolism in AFLD mice is dysregulated, leading to increased hepatic BA synthesis, breaking the balance of BA metabolism, and aggravating disease progression (41). Another recent study found that the changes in gut microbiota caused by long-term ethanol intake may be mediated not by metabolizing ethanol but by the high acetate levels in serum and gut after ethanol metabolism in the liver. The disturbance of gut microbiota may aggravate ethanol-induced liver injury (42). Fortunately, excessive alcohol intake results in low amounts of *Roseburia*, an anaerobe that produces butyric acid, and the supplementation of this bacterium improves the conditions of AFLD in mice (43). Ellagic acid pretreatment significantly decreases the abundance of *E. coli,* increases the abundance of *Lactobacillus* in alcohol-fed mice, and effectively alleviates alcohol-mediated intestinal microbiota dysregulation (44). Oral supplementation with *Akkermansia muciniphila* to compensate for alcohol-mediated reductions in the number of this bacterium has been shown to reduce liver steatosis and damage significantly (45). Astaxanthin also plays a protective role in AFLD by mediating the recovery of abundance of *Akkermansia* (46). The phosphatase complex of clinical value for AFLD has been shown to be partly attributable to the regulatory function of the gut microbiota (47). *Lactobacillus rhamnosus* (LGG) and its culture supernatant combined with immunosuppressive bone marrow mesenchymal stem cells are more effective in the treatment of AFLD mice than the treatment alone (48). These results fully demonstrate that the early supplementation of alcohol-mediated reduction of beneficial bacteria can effectively prevent alcoholic liver disease. Additionally, post-disease supplementation can control further development and deterioration. Bioextracts and biometabolic enzymes also interact with gut microbiota and regulate each other.

### 2.4. Liver fibrosis

Liver fibrosis is primarily characterized by excessive proliferation and deposition of extracellular matrix, a key stage in the development of chronic liver disease to cirrhosis. Blocking or reversing liver fibrosis at this stage will have a good prognosis. In accordance with histopathology, liver fibrosis can be divided into five stages. Intestinal microbiota detection in rat models of liver fibrosis showed that intestinal microbiota is evidently dysregulated, which is manifested as decreased community richness and diversity. Additionally, the intestinal microbiota in each stage is changing (49). In 2021, 217 Hispanics with liver fibrosis are tested for intestinal microbiota, showing the enrichment of various immunogenic commensal bacteria, such as *Prevosa*, and reduced *Bacteroides* and *Enterobacteriaceae* (50). Other studies found a decrease in intestinal *Firmicutes* and *Lactobacilli* and an increase in the probiotic *A. macuciniphila* (51, 52). In recent years, the key steps to block or reverse liver fibrosis are through three aspects: transforming activating hematopoietic stem cells to quiescent state or apoptosis, inhibiting the activation and transformation of hepatic stellate cells, and reducing the concentration of hepatic total BAs (34, 52, 53). Among them, indole-3-carboxaldehyde (3-IALD-MP), a microbial metabolite that blocks liver fibrosis by restoring mucosal integrity with the help of intestinal flora, has been shown to reduce the abundance of proinflammatory *Enterobacter* and increase the abundance of *Lactobacillus reuteri* (54). Studies showed that oral chlorophyll can increase the abundance of *Bacteroidetes,* decrease the abundance of *Firmicutes*, and restore the intestinal microecological balance to reverse liver fibrosis (55). LGG significantly attenuates liver fibrosis. This phenomenon demonstrates that LGG increases the inhibition of BA *de novo* synthesis by activate the FXR-FGF15 signaling pathway and increases the intestinal flora with the ability of secreting bile salt hydrolase to promote BA excretion (56). The dysregulation of intestinal microbiota may lead to the translocation of bacteria or bacterial products, change the hepatic immune microenvironment, promote the release of inflammatory factors, and accelerate the process of fibrosis (51). Liver fibrosis can further promote the dysregulation of intestinal flora, forming a vicious circle. In addition to restoring mucosal integrity and blocking activating signal transduction, the above research results are all involved in cutting off circulation from the dysregulation of intestinal flora.

### 2.5. Cirrhosis

Liver cirrhosis is the terminal stage of various chronic liver diseases, ranging from compensated period to decompensated period, with the most serious consequence being acute/chronic liver insufficiency (ACLF). A study of hospitalized patients with cirrhosis showed that those with dysregulated gut microbiota on admission are more likely to develop ACLF and have higher mortality (57). Another study showed that liver cirrhosis secondary to *Schistosoma japonicum* infection does not have intestinal microbiota dysregulation and has good prognosis (58). This finding indicates that the homeostasis of intestinal flora has a remarkable influence on the prognosis of liver cirrhosis. The metagenomic analysis of patients with cirrhosis has shown a gradual decrease in metagenomic richness and enrichment of foreign bodies as the disease progresses (57). Further studies showed a substantial decrease in beneficial protoresident bacteria, such as butyrate-producing bacteria, and a considerable increase in bacteria associated with mucous layer degradation of the intestinal wall, potential opportunistic pathogens, and pathogenic bacteria in patients with cirrhosis. As the disease progresses, opportunistic pathogens, such as *Pseudomonas dentalis* and *Haemophilus parainfluenzae,* will continue to increase (59). Moreover, liver cirrhosis is positively associated with the degree of bacterial translocation and load of antibiotic resistance genes (ARGs) in the gut microbiota (60, 61). A study used the method of gut microbiota characteristics combined with age analysis to detect cirrhosis accurately and take early intervention measures (62). Another study assessed ACLF and mortality risk by measuring the serum levels of microbial metabolites, such as BAs and aromatic amino acids (63). The development and prognosis of liver cirrhosis are closely related to intestinal microbiota imbalance. The research on correcting intestinal microbiota imbalance and delaying the progression of liver cirrhosis is effective. Dietary cereal and yogurt intakes have been shown to be associated with high gut microbiota diversity and low hospitalization rates in patients with cirrhosis, which is possibly due to increased metabolism of short-chain fatty acids and probiotics (64). The supplementation of lactitol has been shown to reduce the load of pathogenic genes ARGs and VFGs in the intestinal flora; reduce the abundance of pathogenic bacteria associated with liver cirrhosis, such as *Klebsiella*; increase the abundance of beneficial bacteria, such as *Bifidobacterium*; and improve the prognosis of liver cirrhosis (65). Intestinal microbiota dysregulation is improved after transection of the hepatic branch vagus nerve in mice, which may be related to the overgrowth of potentially pathogenic bacteria enhanced by the vagus during cirrhosis (66). For economic and operational reasons, most studies chose stool collection to study intestinal microbiota, which shows no remarkable difference in the diversity of intestinal microbiota in patients with cirrhosis of different etiologies (67). However, colonic mucosal samples collected by rectal swabs and stool samples have different bacterial groups. A classification method characterized by a decrease in the number of *E. coli/Enterobacteriaceae* has been found here (68) and can accurately predict the prognosis of cirrhosis and facilitate appropriate interventions. Therefore, when studying intestinal microbiota, the source of samples should be considered. Sampling methods that are close to the intestinal environment, non-invasive, highly operable, and economically feasible are needed.

### 2.6. Liver cancer

**Figure 1:**
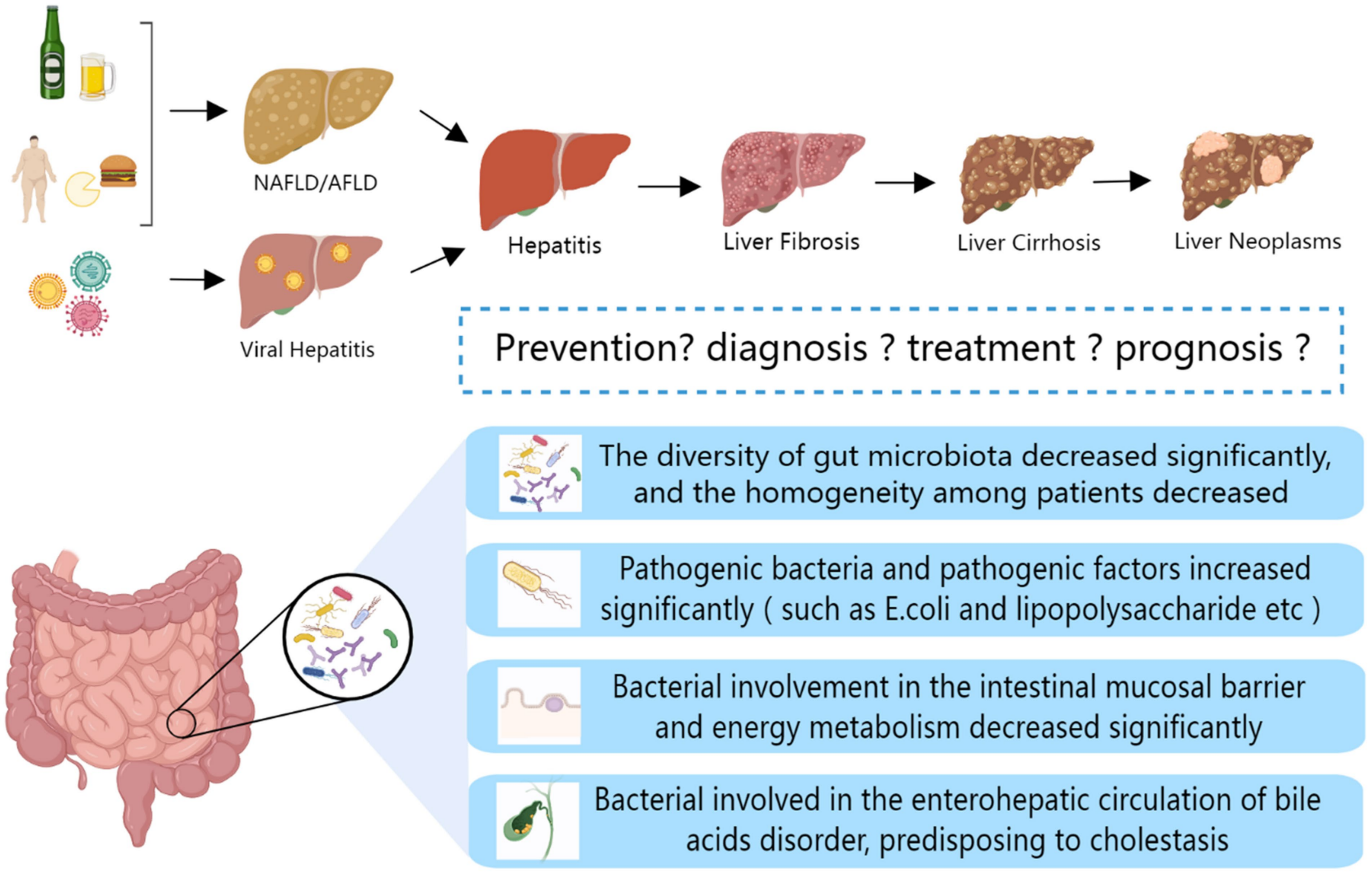
Changes in gut microbiota during liver disease progression.

Hepatocellular carcinoma (HCC) is a malignant tumor occurring in the liver with a high incidence rate and poor prognosis. HCC is easy to be ignored because of inapparent early symptoms. An increase in the abundance of *Bacteroides* and *Ruminants* and a decrease in the abundance of *Akermannii* and *Bifidobacteria* are observed in the intestines of patients with liver cancer (69). Recent studies showed that the dysregulation of intestinal flora aggravates the occurrence of liver cancer, as summarized in Figure 1. With the development and deterioration of HCC, the degree of intestinal flora dysregulation gradually deepens (70). At the same time, studies demonstrated that intestinal microbiota dysregulation leads to the proliferation of colonic epithelial plexus cells; increases the secretion of IL-25; and promotes the migration, invasion, and tumorigenesis of liver cancer cells through activation of M2 macrophages and related pathways (71). The dysregulation of gut microbiota may also promote the progression of HCC by inhibiting CD8 + T cell function and activating regulatory T cells to inhibit the body’s immunity (72, 73). NOD2 acts as a pattern recognition receptor in hepatocytes and interacts with the PAMP of Gram-positive and Gram-negative bacteria. NOD2 has been shown to be overexpressed after the dysregulation of the gut microbiota and is associated with the prognosis of HCC (74). Dietary cholesterol can induce remarkable changes in intestinal flora and metabolites in mice, eventually leading to the formation of NAFLD-HCC. This phenomenon may be related to the dysregulation of intestinal flora and the reduction of probiotics, such as *Bifidobacterium* and BSH-rich bacteria (75, 76). The specific gut microbiota community of patients with HCC and its correlation with markers of inflammatory response make the gut microbiota show remarkable potential for early detection of HCC (77–80). Based on these endless research achievements, scientists have never stopped exploring how to control the further development and metastasis of HCC. Some progress has also been made in the direction of gut microbiota. One team found that c-di-AMP, a metabolite of intestinal microbiota, is likely to cooperate with dsDNA of tumor cells to mediate the CGAS-Sting-IFN-I pathway to control the sensitivity of late radiotherapy in unresectable HCC. Intestinal microbiota, as a new target, can bring new light to patients with radiotherapy resistance (81). In addition, the Chinese patent medicine GanFuLe and the Chinese herbal medicine ginseng have been shown to delay the progression of liver cancer by participating in intestinal microbial metabolism, changing community diversity, and increasing the abundance of beneficial bacteria (82, 83). Ginsenoside Rg3 coupled with nanoparticles can significantly improve intestinal microbiota dysregulation and control the progression of HCC (84). Traditional medicinal resources should not be ignored, we need break down the barrier between traditional and modern medicine to open up a broader space for the research of new anti-liver cancer drugs.

## 3. Gut microbiota and gallbladder principal diseases

### 3.1. Gallstones

Gallstones are characterized by gallbladder and bile duct precipitate stones in the biliary tract system. Gallstones are often divided into cholesterol, bile pigment, and mixed stones in accordance with their chemical properties in clinical practice. For a long time, the biliary tract is thought to be sterile. However, a study in 2013 based on 120 samples, is the first to report the imbalance of intestinal microbiota in patients with gallstones and found that the biliary microbiota is highly similar to the intestinal microbiota and has a high diversity (85). The biliary tract is not completely isolated from the gut. The two are connected through the duodenum and blood, and colonizing the biliary tract is not difficult for intestinal bacteria. Another recent prospective cohort study based on 470,000 participants demonstrated an association between an increased risk of gallstones and disturbances in the gut microbiota caused by the use of stomach acid inhibitors (86). A team found that the intestinal microbiota of mice with gallstones is seriously disordered and identified 33 gallstone markers at the phylum, class, order, family, and genus levels. The paired analysis of liver metabolomics and differential microbiota proved that intestinal microbiota imbalance is a contributing factor to gallstones (87). In addition, the role of intestinal microbiota metabolites as markers of gallstones should not be ignored. For example, trimethylamine is metabolized to TMAO under the action of liver FMO3. Studies proved that high serum TMAO level can promote cholesterol secretion and the formation of gallstones *in vitro* and *in vivo* (88). At the same time, a large number of studies showed that the diversity of intestinal flora in patients with gallstones especially *Firmicutes* is reduced. Considerable changes in multiple functional bacteria are observed. Bacteria secreting the 7α-dehydroxy enzyme increase, and those lowering cholesterol decrease, leading to increased intestinal absorption of BAs and cholesterol (89). In middle-aged and elderly patients, decreased number of bacteria producing prebiotics, such as butyrate and acetate/propionate; decreased number of mucin-degrading bacteria; and increased number of bacteria producing endotoxin are observed. This phenomenon further promotes the disease (90). Another study found abundant *Desulfovibrionales* in the gut microbiota of patients with gallstones and further demonstrated at the animal level that *Desulfovibrionales* increases the hydrophobicity and production of BAs and promotes intestinal absorption of cholesterol and liver secretion to accelerate gallstone formation (91). As mentioned above, the major composition of the biliary microbiota is most likely derived from the gut. A team directly studied the correlation between dietary structure and biliary microbiota in patients with gallstones and found that the abundance of specific microbiota is highly correlated with the intake of dairy products, several fibers, and fatty acids (92). The oral administration of an aqueous extract of the Chinese herb *L. christinae* is shown to improve intestinal microbiota disorder significantly, reduce gallstone formation, and restore histological morphology in mice with gallstones (93). Dietary behavior is highly correlated with intestinal flora and biliary tract flora, further affecting cholesterol and BA metabolism.

### 3.2. Cholangitis

Cholangitis is an inflammation of the bile duct primarily caused by secondary bacterial infection on the basis of cholestasis. Bacteria come from the intestine, blood, and lymph through the duodenal papilla, blood ducts, or lymphatics. A 10-year follow-up cohort study of more than 50,000 participants demonstrated that the use of PPI disrupts gut microbiota balance and is associated with increased risk of cholangitis (94). Decreased intestinal bacterial diversity of patients with primary biliary cholangitis (PBC), decreased abundance of *Clostridium*, increased abundance of *Lactobacillus*, and the intestinal microbiota disorder subsequently affect the metabolism of BAs in the intestinal tract (95). In the same year, another study found that the abnormal BA metabolism of PBC becomes increasingly serious with the progression of the disease and that the level of secondary BA is positively correlated with *Bacteroides stercoris*, *Oscillospira,* and other bacteria enriched in healthy people especially with *B. stercoris*. The reduced degree of abundance of *B. stercoris* can predict the long-term prognosis of patients with PBC (96). The metagenomic sequencing analysis of the gut microbiota in a large number of clinical samples from patients with primary sclerosing cholangitis (PSC) in Norway and Germany showed a remarkable decrease in the species richness of the gut microbiota in patients with PSC. Functionally, the synthesis of essential nutrients vitamin B6 and branched-chain amino acids decreases. Unfortunately, IBD, a common secondary complication, cannot be identified by differences at microbial and metabolite levels (97). A study first found the dysbiosis of fungal flora in the gut of patients with PSC in 2022, which is manifested by increased diversity and a change in composition ratio and bacterial–fungal interaction relative to bacteria (98). IgG4-associated sclerosing cholangitis also exhibits decreased gut microbiota diversity and changes in structural proportions as PSC, but the microbe–metabolite relationship is quite different from PSC (99). Although evidence showed that the intestinal microbiota disorder exists in patients with cholangitis, the specific pathogenic mechanism should still be explored to strengthen the evidence and open up the path for more treatment methods. In PSC mouse models, intestinal microbiota dysregulation is found to stimulate liver injury by activating NLRP3 inflammasomes through toxin translocation (100). Compared with the direct pathway of bacterial translocation, the indirect pathway of intestinal microbiota affecting BA metabolism has made substantial progress in recent years. In genetic PSC mouse models, intestinal microbiota is found to increase BA synthesis in reverse by inhibiting the FXR signaling pathway, further damaging the bile duct and liver. Serum C4 level was identified as a potential prognostic reference of PSC (101). Moreover, a study directly identified six essential enzymes for the conversion of primary BAs to secondary BAs by gut microbiota and successfully produced DCA and LCA by introducing relevant operon genes into *C. sporogenes*, which are confirmed in germ-free mice (102). Bacterial translocation and BA metabolism disorders are the main pathways for intestinal flora to participate in the pathogenic process. Detailed studies are needed in the future to provide new targets and ideas for the treatment of cholangitis.

### 3.3. Cholangiocarcinoma

Cholangiocarcinoma is a malignant tumor occurring in the biliary tract system. In accordance with different anatomical sites, cholangiocarcinoma is often divided into intrahepatic (ICC) and extrahepatic cholangiocarcinoma and is primarily manifested by the difficulty of early diagnosis, rapid progression, and high degree of malignancy. The biliary tract is closely related to the intestine. Whether the intestinal microbiota can be used as a specific biomarker for early screening and progression prediction of cholangiocarcinoma has been attracting much attention. As early as 2013, hamsters infected with carcinogenic liver flukes *Opisthorchis viverrini* are found to have increased diversity of intestinal microbiota and abundance of *Trichospiraceae*, *Ruminococcaceae*, and *Lactobacillaceae* and decreased abundance of *Porphyromonas*, *Danthiaceae*, and *Eubacteraceae* (103). Subsequently, high abundance values of *Bifidobacteriaceae*, *Enterobacteriaceae,* and *Enterococcaceae* especially Bifidobacteriaceae are found in the intestinal microbiota of patients with clinical cholangiocarcinoma and infected with *O. viverrini* (104). In 2019, the disorder of biliary flora in patients with extrahepatic cholangiocarcinoma is reported for the first time, and the dominant bacterial phyla of ECC’s bile flora are *Proteobacteria*, *Firmicutes*, *Bacteroidetes*, and *Actinobacteria* (105). The intestinal microbiota diversity of ICC increases, and the abundance values of *Lactobacillus*, *Actinomyces*, *Peptostreptococcaceae*, and *Alloscardovia* significantly increase at the genus level especially *Lactobacillus*. Alloscardovia has a strong positive correlation with the serum fecal ratio of tauroursodeoxycholic acid, an ICC BA marker. The homogeneity of gut microbiota (β-diversity) among ICC populations decreases, and further reduction of β-diversity and increased abundance of *Ruminococcaceae* suggest the risk of vascular invasion (VI). The prognosis after VI is poor, and the 3-year survival rate is remarkably reduced. The abundance of *Pseudoramibacter* is positively correlated with survival time (106). Previous studies showed that patients with cholangiocarcinoma have a significantly higher detection rate of *H. pylori* in the gut and biliary tract than patients with benign lesions of the bile duct and that the relatively noninvasive detection rate of *H. pylori* in the gut is not specific (107, 108). In 2021, a study found that compared with that in healthy people, the composition of intestinal microbiota in patients with cholangiocarcinoma is significantly different at the genus level. Thus, statistical analysis is used to establish a specific microbiota model B-F-R (*Burkholderi*a–*Caballeronia*–*Paraburkholderia*, *Faecalibacterium*, and *Ruminococcus*) for the early diagnosis of CCA (109). In the same year, another study found that intestinal barrier is damaged in patients with cholangiocarcinoma, and Gram-negative bacteria enter the liver to induce CXCL1 expression in hepatocytes through a TLR4-dependent mechanism, further leading to the accumulation of CXCR2+ polymorphonuclear myeloid-derived suppressor cells. This sequence of reactions inhibit the immune system of anti-tumor immunity and exacerbating the process of cholangiocarcinoma (110). The specific mechanism of intestinal microbiota in the disease process of cholangiocarcinoma is minimally studied and needs to be further explored.

## 4. Biotherapies

### 4.1. Antibiotics

Antibiotics play an important role in preventing secondary infection and poor prognosis in major diseases of liver, gallbladder, and pancreas. However, antibiotic treatment may reduce the diversity of intestinal flora and promote the accumulation of antibiotic-resistant bacteria and drug-resistant genes by acting on the bacteria themselves or the intestinal environment (111). To adjust prescriptions and monitor the emergence of resistance, the culture and sensitivity results of the samples obtained should be reviewed regularly when antibiotics are used. However, new antibiotics or adjuvants to enhance the sensitivity of bacteria to antibiotics are needed in clinical practice due to the endless emergence of drug-resistant bacteria. In 2021, a follow-up study of 1,175 patients with decompensated cirrhosis showed that the proportion of patients with acute and chronic liver failure secondary to bacterial infection reaches 48% before and after admission. This proportion varies with geographic region, sex, age, and history of liver disease. The most troublesome is drug-resistant bacterial infections, which are difficult to solve due to the lack of corresponding antibiotics in clinical practice (112). Similarly, the clinical management of acute cholangitis is primarily based on source management and empirical antibiotic treatment (third-generation cephalosporins, piperacillin/tazobactam, and flunolones) to avoid secondary bacterial translocations and iatrogenic infections. A survey study showed that according to current empirical treatment, only 51% of patients’ bile culture bacteria and 69% of patients’ blood culture bacteria can be covered. The bacterial coverage of the current empirical antibiotic treatment population needs to be improved, but piperacillin/tazobactam can still maintain 100% coverage of bacterial infections in patients with community-acquired cholangitis who are not treated with indwelling bile duct drainage and ICU treatment (113). Empirical antibiotic treatment requires large samples and data to be discussed and analyzed.

### 4.2. Probiotics, prebiotics, postbiotics, and synbiotics

Probiotics are selected from the dominant species of normal flora and transplanted into the patient’s body to play their inherent physiological role. Prebiotics are substances that can be selectively utilized by host microorganisms to benefit host health. Postbiotics are a variety of metabolites after fermentation and processing of probiotics, including short-chain fatty acids (SCFAs), functional proteins, cell lysates, etc. Synbiotics are mixture of microorganisms and their substrates that promote the health of the host organism. Probiotics, prebiotics, postbiotics, and synbiotics intervene in major diseases of liver, gallbladder, and pancreas by regulating intestinal flora, regulating BA synthesis, anti-inflammatory functions, repairing intestinal mucosal barrier, and other ways. Intervention of lithogenic-diet (LD)-fed mice with *Lactobacillus reuteri* and *Lactobacillus plantarum* regulated the gut microbiota, which increased the relative abundance of *Muribaculaceae* and *Akkermansia* in the former and latter, respectively. These strains may alleviate gallstones and hepatic steatosis by activating FXR and inhibiting the activities of hepatic sterol 7α-hydroxylase (CYP7A1) and hepatic sterol 7α-hydroxylase (CYP7B1) (114). The probiotic *Prevotella copri* can significantly increase the intestinal microbiota diversity and activate the FXR-Cyp7a1 signaling pathway to inhibit BA synthesis to improve the progression of cholestasis and liver fibrosis in PSC mouse models (115). In the mouse model of PSC, the intestinal microbiota metabolite 3-IALD is demonstrated to repair the intestinal mucosal barrier by activating the AhR/IL-22 signaling pathway, significantly reduce bacterial translocation and total bacterial abundance in liver, and protect liver to reduce inflammation and fibrosis (54). In the mouse model of alcoholic liver disease, the combination treatment of aged garlic extract and *Lactobacillus rhamnosus* can reduce intestinal oxidative stress and inflammation, regulate intestinal flora, promote the repair of intestinal mucosal barrier, and inhibit the further deterioration of the disease (116). Polylactose, a new prebiotic, was found to reduce obesity and liver lipid and cholesterol levels in high fat-diet-fed rats. The possible mechanism of action is to increase the relative abundance of *Bifidobacterium*, reduce intestinal PH and regulate intestinal flora (117). LAB strains (*Lactiplantibacillus plantarum*, *Lactobacillus brevis*, and *Weissella cibaria*) pretreatment of human hepatocytes significantly inhibited the TGF-β/SMAD signaling pathway. It also reduced the collagen deposition, cell autophagy and apoptosis (118). The experimental phenomena observed fully illustrate the potential of LAB strains to reverse liver fibrosis. In conclusion, probiotics, prebiotics or postbiotics can play multiple roles, such as signaling molecules and regulatory factors, in addition to regulating intestinal flora. Before their huge application potential, we also have to face unknown security risks, and more complex mechanisms need to be explored in the future.

### 4.3. Fecal bacteria transplantation

Fecal microbiota transplantation is an effective means to reconstruct the intestinal flora. The functional microbiota of healthy people is separated and purified by laboratory instruments and transplanted to the gastrointestinal tract of patients to achieve the effect of regulating and reconstructing the intestinal flora. Fecal microbiota transplantation (FMT) initially gained worldwide attention due to its promising efficacy in the treatment of refractory Clostridium difficile infection. At present, there are many studies on fecal microbiota transplantation in intestinal diseases such as inflammatory bowel disease and irritable bowel syndrome and nervous system diseases such as autism, but the research on hepatobiliary and pancreatic diseases is not extensive. A 90-day clinical study compared prednisolone with fecal microbiota transplantation in the treatment of severe alcoholic hepatitis. It was found that the functional scores were similar to those of prednisolone, the secondary infection rate was much lower than that of prednisolone, and the 28-day survival rate, especially the 90-day survival rate was significantly higher than that of prednisolone. This may be related to the fact that fecal microbiota transplantation slowly increases the diversity of gut microbiota to establish new communities (119). The structure of gut microbiota is highly related to the outcome of HBV infection, which is expected to reduce the immune tolerance of liver and the chronic infection rate of hepatitis B virus by intestinal flora transplantation (15). In 2019, a clinical trial of 10 patients with PSC undergoing FMT treatment demonstrated the safety of FMT in patients with PSC for the first time. In all patients, the diversity of gut microbiota increases in the first week of treatment, and the diversity is negatively correlated with serum ALP level, proving effectiveness to some extent (120). Fecal bacteria transplantation donors are not limited to others. Another study on autologous transplantation isolated and purified *E. coli* from the mice’ own feces, which are added with functional genes and re-transplanted into mice. Surprisingly, these bacteria can achieve long-term survival or even lifelong colonization in the intestinal tract of mice (121). Given that the lifestyle and eating habits of primitive people have not been affected by industrial society, some people even propose to transplant the intestinal flora of primitive people (122). However, the author believes that the current environment can no longer adapt to the original bacteria after tens of thousands of years of evolution. A study recently successfully constructed a synthetic microbial community with clear composition, high complexity, and effective reflection of the role of human gut microbiota. This study provides a new material basis for the selection of fecal bacteria transplantation donors (123).

### 4.4. Drugs that directly regulate short-chain fatty acids

Carbohydrates and dietary fiber are metabolized into short-chain fatty acids under the action of intestinal flora, it is mainly butyrate, propionate and acetate. SCFAs increase intestinal absorption area, regulate intestinal flora, and serve as an important energy source for intestinal epithelium. Butyrate with the prominent ability of immune function regulation also provides material basis for its anti-inflammatory and antitumor properties. Sodium butyrate intervention in NAFLD mice induced by high-fat diet can inhibit fat synthesis, reduce steatosis and improve liver function by activating LKB1-AMPK-Insig (insulin-inducible gene) signaling pathway (124). Another similar study found that sodium butyrate may also upregulate miR-150 expression which negatively target CXCR4 to delay the progression of NAFLD (125). HBV-encoded oncoprotein HBx promotes the progression of hepatocellular carcinoma by changing host gene expression and multiple pathway activity. Researchers found that feeding HBx transgenic mice with SCFAs for 3 months inhibited the development of chronic hepatitis B to hepatocellular carcinoma and significantly reduced hepatocellular carcinoma nodules. It may down-regulated HBx activation pathway and reduced the viability of HBx-transfected cell lines (126). In the comparison between FXR knockout mice and control mice, butyrate also reduced hepatic β-muricholic acid (β-MCA) and bacteria-generated deoxycholic acid (DCA), and eliminated liver lymphocyte infiltration, which was beneficial to the prognosis of hepatitis (127). Sodium butyrate intervention in lithogenic-diet (LD)-fed mice reduced the incidence of gallstones by 75%. Further studies showed that sodium butyrate reshaped the gut microbiota of mice, activated the FXR-FGF-15/SHP signaling pathway, and inhibited bile acid synthesis. It increased the levels of functional proteins related to bile acid metabolism and promoted the reabsorption and excretion of bile acids in the intestine (128). It plays a protective role in hepatobiliary diseases, whether increasing content of SCFA is added directly or indirectly by intestinal flora. On the one hand, SCFAs may act on the intestinal mucosal barrier to inhibit bacterial translocation. On the other hand, SCFAs may reach the corresponding organs through the gut-liver-biliary axis to activate associated signaling pathways, and then play an anti-inflammatory and anti-tumor role. However, it is noteworthy that soluble dietary fiber inulin——one of the sources of SCFAs, can induce icteric HCC in experimental mice after prolonged feeding. Other soluble dietary fibers, including pectin, showed the same adverse results, whereas Non-fermentable or Insoluble Fiber Cellulose did not (129). The supplement of SCFAs is not as “No harm but good” as we think, and more in-depth thinking and research on the source of SCFAs are needed in the future.

### 4.5. Others

**Figure 2:**
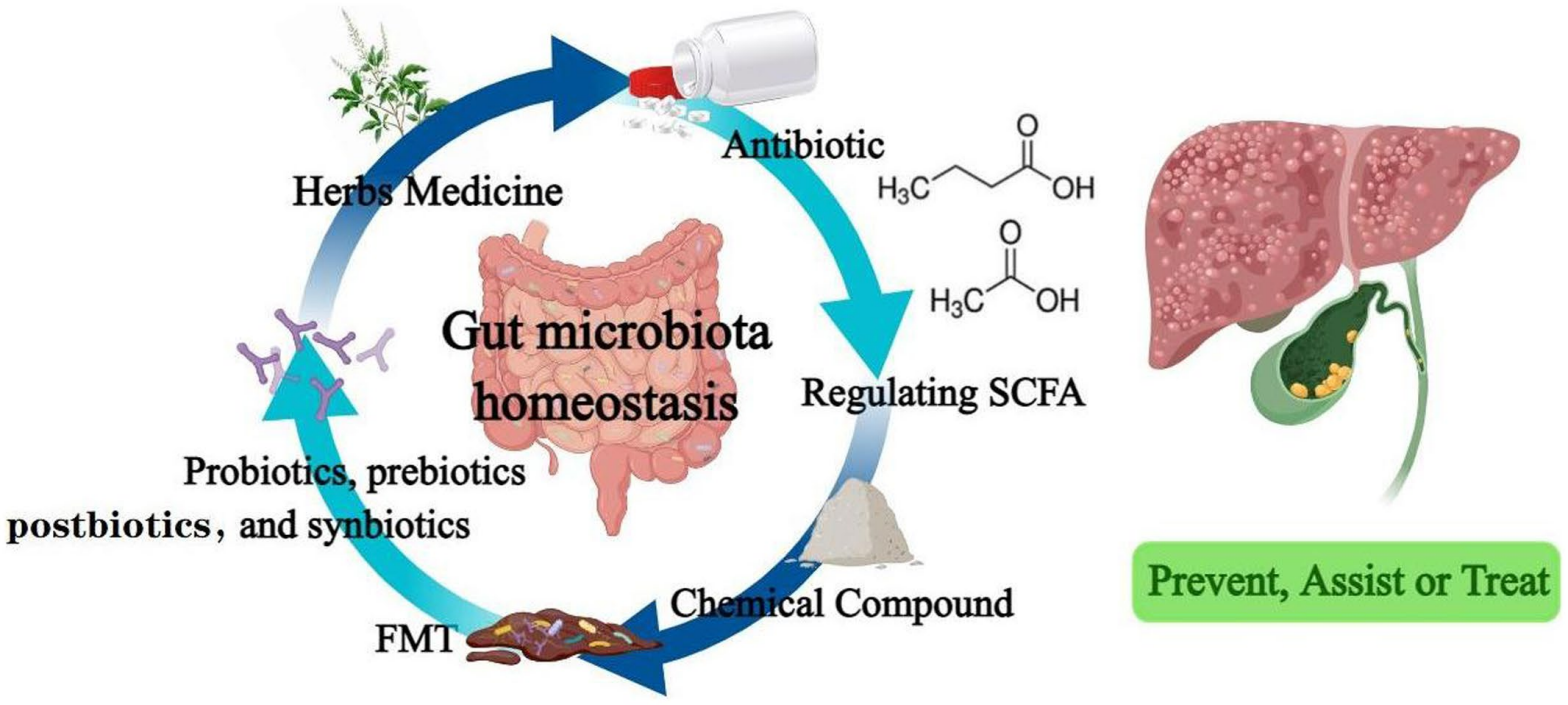
Drugs that regulate intestinal flora effectively prevent, assist or treat hepatobiliary diseases.

In addition to those summarized above (Table 1), traditional Chinese medicine prescription and their source components have made outstanding contributions to liver and gallbladder diseases. Si-Wu-Tang, a traditional Chinese medicine used in the treatment of gynecological diseases, has been shown to improve and stabilize intestinal microbiota and significantly reverse the disease course in liver fibrosis mouse model (130). The organic compound cholestyramine has also been found to alleviate cholestasis possibly by increasing the abundance of *Lachnospiraceae,* reducing the abundance of *Klebsiella pneumoniae* in the gut of patients with primary cholangitis, and blocking BA reabsorption (131). Clinical application of antibiotics is in a difficult position due to the existence of drug-resistant bacteria and bacterial biofilms. Administration of a supernatant derived from nanoscale iron sulfide (nFeS supernatant) demonstrated excellent antimicrobial and biofilm destruction capabilities *in vitro*. In mice with cholecystitis accompanied by gallstones, it can not only play the role of sterilization, treatment and removal of gallstones. It can also regulate bile acid metabolism and prevent the formation of gallstones (132). This highly biocompatible chemical is expected to be a potential treatment alternative to antibiotics and cholecystectomy. Figure 2 summarizes all of biotherapies described in this paper.

**Table 1:** Registered clinical trials and animal studies about biotherapy to treat liver and gallbladder disease.

Biotherapy	Resource	Condition or disease	Outcomes	Mechanism of action	References
*Limosilactobacillus reuteri* strain CGMCC 17942、*Lactiplantibacillus plantarum* strain CGMCC 14407	Male C57BL/J mice(About 8-Week-old)	CGS	Alleviate gallstones and hepatic steatosis	Regulate the gut microbiota,which increased the relative abundance of Muribaculaceae and Akkermansia in the former and latter, respectively. Activate FXR and inhibit the activities of CYP7A1 and CYP7B1	Ye et al. (114)
*Prevotella copri*	Male C57BL/J mice(7-Week-old)	PSC	The progression of cholestasis and liver fibrosis was improved	Increase the diversity of intestinal flora and activate the FXR-Cyp7a1 signaling pathway to negatively inhibit the synthesis of bile acids	Jiang et al. (115)
3-IALD	C57BL/6 mice(6–8-week-old)	PSC	Protect the liver, reduce inflammation and fibrosis	Activate the AhR/IL-22 signaling pathway and reduce bacterial translocation	D’Onofrio et al. (54)
A combination of aged garlic extract (AGE) and *Lactobacillus rhamnosus* MTCC1423	Male Wistar rats(8-week-old)、Caco-2 cells	ALD	Inhibit the further deterioration of ALD	Reduce intestinal oxidative stress and inflammation， promote the microbiome’s shift toward Firmicutes and the repair of intestinal mucosal barrier	Patel et al. (116)
Polylactose	Male Wistar rats (4-5-week-old)	NAFLD	Reduce obesity and liver lipid and cholesterol levels in high fat-diet-fed rats	Increase the relative abundance of *Bifidobacterium*, reduce intestinal PH and regulate intestinal flora	Soares et al. (117)
LAB strains (*Lactiplantibacillus plantarum, Lactobacillus brevis,* and *Weissella cibaria*)	Human hepatic stellate cell line (LX-2)	Hepatic fibrosis	Reduce the collagen deposition, cell autophagy and apoptosis	Inhibit the TGF-β/SMAD signaling pathway	Kanmani and Kim (118)
FMT	120 participants	SAH	Compared with Prednisolone, reduce secondary infection rate and improve 90-day survival rate	Increase the diversity of gut microbiota to establish new communities	Pande et al. (119)
FMT	BALB/c and C57BL/6 mice(6-8-week-old)	Hepatitis B	Reduce the immune tolerance of liver and the chronic infection rate of hepatitis B virus	Reconstitution of the gut microbiota	Wang et al. (15)
FMT	10 participants	PSC	The first study to demonstrate that FMT in PSC is safe	Increase the diversity of gut microbiota	Allegretti et al. (120)
Sodium butyrate	Male C57BL/6 mice(6-week-old)	NAFLD	Reduce steatosis and improve liver function	Activate LKB1-AMPK-Insig (insulin-inducible gene) signaling pathway to inhibit fat synthesis	Zhao et al. (124)
Sodium butyrate	Male C57BL/6 mice(8-week-old)	NAFLD	Delay the progression of NAFLD	Upregulate miR-150 expression to negatively target CXCR4	Zhang et al. (125)
The sodium salts of butyrate (202410), propionate (P1880) and acetate (S2889).	C57BL/6 mice	Hepatitis B	Inhibite the development of chronic hepatitis B to hepatocellular carcinoma	Down-regulate HBx activation pathway and reduce the viability of HBx-transfected cell lines	McBrearty et al. (126)
Sodium butyrate	C57BL/6 mice	Hepatitis	Reduce hepatic β-muricholic acid (β-MCA) and bacteria-generated deoxycholic acid (DCA), and eliminated liver lymphocyte infiltration	Reverse dysregulated BA synthesis and its associated hepatitis.	Sheng et al. (127)
Sodium butyrate	C57BL/6 mice(8-week-old)	CGS	Reduce the incidence of gallstones by 75%	Reshape the gut microbiota, activate the FXR-FGF-15/SHP signaling pathway, and increase the levels of functional proteins related to bile acid metabolism to promote the reabsorption and excretion of bile acids in the intestine	Ye et al. (128)

CGS, cholesterol gallstone; PSC, primary sclerosing cholangitis; ALD, alcoholic fatty liver; NAFLD, non-alcoholic fatty liver disease; FMT, fecal microbiota transplant; SAH, severe alcoholic hepatitis.

## 5. Summary and conclusion

As the largest microecosystem in symbiosis with the human body, the gut microbiota and the host eat and live together, and their fate is closely related. In this review, we discussed the changes of intestinal flora and its metabolites, the metabolic and signaling pathways in which it involved in the occurrence and development of liver and biliary diseases. Moreover, we summarized the biologic therapies that mediate intestinal flora to improve diseases, including antibiotics, probiotics, prebiotics, postbiotics and synbiotics, fecal bacteria transplantation, and drugs that directly regulate SCFAs, etc. We found that intestinal flora, in addition to playing a role in the treatment and improvement of the prognosis of liver and bile diseases (even when the tumor is resistant to chemotherapy), can also play a role as a biomarker in the early stage of disease, which can improve the prediction accuracy of chronic diseases to achieve the preparation for prevention in advance. Therefore, we have enough reason to believe that intestinal flora will show a huge potential in the future clinical application of tertiary prevention of hepatobiliary diseases.

## Author contributions

XH, YY, XL, XZ, DL, YM, MZ, XC, and BZ: conceptualization, discussion, and original draft preparation. DD, CY, and YL: supervision and manuscript reviewing and editing. All authors contributed to the article and approved the submitted version.

## Funding

This work was partly supported by the National Nature Science Foundation of China (No. 81860653 and 82060654); Foundation of Key Laboratory of noncoding RNA and drugs in Universities of Sichuan Province (FB20-02); National Innovation and Entrepreneurship Training Program for College Students (S202110719125 and 202210719026).

## Conflict of interest

The authors declare that the research was conducted in the absence of any commercial or financial relationships that could be construed as a potential conflict of interest.

## Publisher’s note

All claims expressed in this article are solely those of the authors and do not necessarily represent those of their affiliated organizations, or those of the publisher, the editors and the reviewers. Any product that may be evaluated in this article, or claim that may be made by its manufacturer, is not guaranteed or endorsed by the publisher.
